# Navigating maternity health care: a survey of the Canadian prairie newcomer experience

**DOI:** 10.1186/1471-2393-14-4

**Published:** 2014-01-06

**Authors:** Zubia Mumtaz, Beverley O’Brien, Gina Higginbottom

**Affiliations:** 1 School of Public Health, University of Alberta, 3–309 Edmonton Clinic Health Academy, 11405 – 87 Ave, Edmonton, AB T6G 1C9, Canada; 2 Faculty of Nursing, University of Alberta, 3–309 Edmonton Clinic Health Academy, 11405 – 87 Ave, Edmonton, AB T6G 1C9, Canada

**Keywords:** Maternity services, Prairie Provinces Canada, Newcomer experiences, Maternity experiences survey

## Abstract

**Background:**

Immigration to Canada has significantly increased in recent years, particularly in the Prairie Provinces. There is evidence that pregnant newcomer women often encounter challenges when attempting to navigate the health system. Our aim was to explore newcomer women’s experiences in Canada regarding pregnancy, delivery and postpartum care and to assess the degree to which Canada provides equitable access to pregnancy and delivery services.

**Methods:**

Data were obtained from the Canadian Maternity Experiences Survey. Women (N = 6,241) participated in structured computer-assisted telephone interviews. Women from Alberta, Saskatchewan and Manitoba were included in this analysis. A total of 140 newcomers (arriving in Canada after 1996) and 1137 Canadian-born women met inclusion criteria.

**Results:**

Newcomers were more likely to be university graduates, but had lower incomes than Canadian-born women. No differences were found in newcomer ability to access acceptable prenatal care, although fewer received information regarding emotional and physical changes during pregnancy. Rates of C-sections were higher for newcomers than Canadian-born women (36.1% vs. 24.7%, p = 0.02). Newcomers were also more likely to be placed in stirrups for birth and have an assisted birth.

**Conclusion:**

Although newcomers residing in Prairie Provinces receive adequate maternity care, improvements are needed with respect to provision of information related to postpartum depression and informed choice around the need for C-sections.

## Background

Maintaining health equity is a challenge facing most western democracies as they respond to changing patterns of immigration. Within the G8, Canada is one of the key receiving countries for newcomers, accepting an average of 250,000 immigrants annually [1]. Further, along with most western democracies, Canada is a country of increasing ethno-cultural diversity and subsequently faces challenges around equitable distribution of resources and opportunities to the many very disparate communities across the country [2]. Access to health care and the health status of newcomer populations is one such important equity issue [3].

According to the 2011 Canadian census, increasing numbers of these newcomers are settling in the Prairie Provinces of Alberta, Saskatchewan and Manitoba. For example, 10 of the 15 census clusters with the highest population growth were situated in Alberta. Saskatchewan had a strong increase in its population growth, increasing from minus 1.1% between 2001 and 2006 to plus 6.7% between 2006 and 2011. Population growth has doubled in Manitoba since 2006. Much of the population increase in these three provinces is attributed to increases in both immigration and inter-provincial migration [1].

The three countries of origin for most newcomers to the Prairie Provinces of Canada are India, China and the Philippines. Other countries contributing newcomers to the prairies include Sudan, Somalia, Democratic Republic of Congo, Sierra Leone, Liberia, Afghanistan, Mexico and Iraq [4]. Ranging in age from 25 to 44 years old with an average of 29.8 years, newcomers tend to be young [5]. This is the peak reproductive age in need of high quality maternity care. The ability to provide culturally appropriate and safe maternity care is thus a key consideration for provincial health policy makers and providers. Because of the vast cultural, ethnic and background diversity of the newcomers, their expectations for maternity care will vary widely [6]. For example, women from parts of some countries in East Africa routinely undergo female genital cutting, often referred to as female circumcision; this will continue to affect their expectations and needs for culturally competent maternity care as they migrate to other areas [7]. For newcomer Punjabi women who migrate from India, deeply embedded traditional health beliefs and practices during the perinatal period; involvement of family members and previous relationships with health care providers in their home country mediate their maternity care expectations in Canada [8]. While articulation of newcomer maternity experiences for policy decisions is relatively new in Canada, recent national guidelines do explicitly refer to population diversity and the need for maternity providers to tailor services to the needs of those they serve [9]. As a component of health care, maternity care is a provincial responsibility but to receive federal monetary disbursements all provinces and territories must comply with the Canada Health Act, 1984. There is considerable inter-provincial variation in service organization although, for most part, there is agreement on national care standards. These include the importance of ‘family-centered’ guidelines that emphasize birth as a normal developmental process and the importance of social support, informed choice and respect [9].

There is evidence that many newcomers in western industrialized countries struggle to navigate health systems, including maternity care services [10,11]. Language issues have been identified as one of the key challenges [12]. This results in superficial and inadequate initial communication and primary assessments between caregiver and recipient [6]. Such unsatisfactory therapeutic encounters may result in multiple consultations or failure of a newcomer to understand or comply with care plans or treatment [13]. They may also receive unnecessary interventions such as C-sections, thereby increasing personal risk and wasting limited resources [14]. All of these encounters can result in newcomers being less satisfied with and less willing to utilize services leading to sub-optimal outcomes [15].

To facilitate equitable service delivery to all women requiring maternity care within the Canadian Prairie Provinces, there is a need to understand the range of experiences and the inter-play of influences that shape newcomer women’s maternity experiences. We propose to document the maternity care seeking experiences of these women and reveal any difference in access and use compared with Canadian-born women. Specifically we aim to answer the following questions: How do newcomer women to the Canadian Prairies experience maternity services? In particular, how did they navigate the health system and how satisfied were they with the care they received? An understanding of these issues will help to positively influence future directions for maternity services delivery to newcomer women and help to inform the current evidence-base for addressing inequities in care.

## Methods

Data were obtained from the Canadian Maternity Experiences survey MES conducted by a working group of the Canadian Perinatal Surveillance Systems (CPSS), Public Health Agency of Canada and Statistics Canada. This survey sample consists of women who gave birth in Canada within three months prior to the 2006 Census. Women who reported during the 2006 Canadian Census that they gave birth in Canada within the previous three months were eligible for inclusion in the (MES). From an estimated 75,863 eligible women, 8,542 were randomly selected to participate in a structured computer-assisted telephone interview administered in three official languages (English, French, Inuktitut) by trained female Statistics Canada interviewers. Interviews were also conducted in 13 additional most-commonly spoken languages by interviewers who were fluent in those languages and had access to the translated glossaries of terms.

Women were stratified by province or territory where they gave birth, rural or urban residence and age (<20 or >20) to ensure representation of vulnerable groups. Women were eligible to participate in the survey if they were at least 15 years of age, gave birth in Canada to a singleton live infant, and were living with their infant at the time of the interview. The interviews took place when infants were between 5 and 10 months old in the provinces (96.9%) and 9 to 14 months old in the territories (3.1%). For logistical reasons women were excluded if they were living on First Nations reserves or in institutions at the time of the survey. Properties of the MES survey instrument are published elsewhere [16]. Although most women were willing to participate, the useable response rate was 78% (n = 6,421) with most mothers (96.9%) being interviewed between 5 and 9 months postpartum [17]. The research protocol was reviewed by Health Canada’s Science Advisory Board and Research Ethics Board, and the Federal Privacy Commissioner. Approval was received from Statistics Canada’s Policy Committee prior to implementation.

The population of interest for this manuscript is limited to those in the MES who gave birth in one of the three Prairie Provinces. Newcomers were defined as women who arrived in Canada after 1996 and those in the comparison group were Canadian born. A total of 140 newcomer and 1137 Canadian-born women met these criteria. Newcomers included landed immigrants, refugees, students, visitors and temporary workers. Variables of interest selected from the MES include indicators of socio-demographic status, maternal perceptions of timely receipt of services, continuity of care, type of provider, opportunities for informed choice and degree of medical interventions. Questions that assessed maternal levels of satisfaction with maternity services as well as information received were also included. We chose to focus on Alberta, Saskatchewan and Manitoba for two reasons: this study is part of and will contribute data to a larger mixed method project where the focus is to strengthen maternal health among newcomers in the Prairie Provinces and because collectively, these Prairie Provinces are attracting the greatest numbers of newcomers to Canada [18]. Although there is some intra-provincial diversity in the geographic, political and social make up that potentially has consequences for their healthcare systems, there are more commonalities than differences.

The statistical package Stata 12 was used to conduct secondary analysis of relevant MES data. For each variable, weighted proportions were calculated using survey sample weights where the weighted sample represented 75,863 women. Bootstrap weighing was not done for the Prairie Provinces sample because the size was insufficient. Therefore the design effect has not been taken into account in calculating the estimators. Weighted proportions were calculated using Pearson’s Chi Square test with 95% confidence intervals. Statistical significance for all analyses was set at *p* < 0.05. At our request, Statistics Canada released data for this study through the Research Development Centre (RDC) at the University of Alberta. The RDC was responsible for ensuring anonymity for all reported findings. For this reason we were required to suppress any findings where numbers were very small. This requirement also supported the validity of findings in that if we reported findings with a very small sample, we could possibly be reporting atypical results.

## Results

Socio-demographically, newcomer women are significantly more likely to be to be married (96% vs. 70%) and older (17% vs. 13.5% were more than 35 years of age at last birth) than Canadian-born women. They are more educated, with 55% reporting a university education compared to 26% of Canadian-born women and more likely to report a lower income. (See Table 1).

**Table 1:** Comparison of Demographic Characteristics on Newcomer women and Canadian-born women using weighted proportions

	**Newcomer**	**Canadian-born**	
	**(N = 140)**	**(N = 1137)**	
**Demographic characteristics**	Weighted%	Weighted%	p-value*
**Maternal age at birth**			0.01
<19 years	Suppressed*	5.3	
20–34 years	82.6^a^	81.2	
> - 35 years	17.4	13.5	
**Maternal education**			0.00
High school or less	18.6	26.9	
Some post-secondary or trades	26.9	47.4	
University degree	54.5	25.7	
**Marital status**			0.00
Currently married^b^	Over 96	70.3	
Not married	Suppressed*	29.7	
**Household income**			0.01
Less than 30 K	4.7	8.32	
30,000-49,999	29.7	17.0	
50,000-79,999	45.0	41.9	
80,000 +	20.6	32.9	
**Region of birth**			
America	16.2		
Europe	18.4		
Africa	7.7		
Asia + Oceania	56.7		
**Place of residence**			0.02
Urban	92.8	86.7	
Rural	7.2	13.3	
**Parity**			0.26
Primipara	50.7	44.9	
Multipara	49.3	55.1	

*findings suppressed when sample size is insufficient to guarantee anonymity.

^a^The age groups were categorized differently because of privacy concerns related to small sample size in newcomer women (34 years & below).

^b^Either married or live in common law.

In contrast to the differences in socio-demographic characteristics, no differences were reported between newcomer and Canadian-born women with respect to their ability to navigate the health care system. Both groups of women were equally likely to time prenatal visits when they wanted them, have the same provider throughout pregnancy and birth, and have contact from the Public Health Nurse after birth. Although statistically significant, between-group differences in the number of prenatal visits (11.5 vs. 12.8) favored the Canadian-born group but could not be considered clinically important in that both groups received an adequate number of visits. Similarly there were no between-group differences in information that women received regarding what to expect during pregnancy and childbirth, with the exception of information about physical and emotional changes. In these cases fewer newcomers reported receiving this information (87%) than did their Canadian-born counterparts (95%, p < 0.001).

Newcomer women were less likely to report receiving information about postpartum depression, breastfeeding, birth control and Sudden Infant Death Syndrome (See Table 2). While both groups of women reported equal rates of health care provider recommendations for C-section, newcomer women were significantly more likely to actually have a C-section (36.1% vs. 24.7%, p = 0.02) (Table 3). Newcomer women were also significantly more likely than Canadian-born women to report they were put in stirrups for their birth and that their baby was delivered with forceps.

**Table 2:** Comparison of experiences of navigating the health system by newcomer and Canadian-born women using weighted proportions

	**Newcomer**		**Canadian-born**		**P-value**
	**N = 140**		**N = 1137**		
	**Weighted mean**	**95% CI**	**Weighted mean**	**95% CI**	
**Navigating health system variables**					
Timing of pre-natal visit (mean no. of weeks)	7.46 weeks	6.75, 8.17	7.13 weeks	6.92, 7.35	0.83
	Weighted %		Weighted %		
Pre-natal care as early as she wanted					0.97
*Yes*	87.7	81.1,92.3	87.5	85.4,89.4	
Attended prenatal classes					0.45
*Yes*	32.4	24.7,41.1	34.1	31.3,37.1	
Number of prenatal visits	11.5	10.9, 12.1	12.8	12.5, 13.0	0.002
**Continuity of care**					
Had the same provider at pregnancy & birth					0.20
Yes	48.7	40.1,57.3	47.5	44.5,50.6	
Preferred to have the same provider					0.46
Yes	48.0	36.1,60.1	44.1	40.0,48.4	
Husband was present during labour					0.39
Yes	93.0	87.4,96.2	90.6	88.7,92.2	
A companion was present during labour					0.15
Yes	32.9	25.2,41.5	39.2	36.2,42.2	
**Communication and information exchange**					
Information provided about physical changes to body					0.001
Yes	87.0	79.6,91.9	95.0	93.6,96.2	
Information provided about emotional changes					0.006
Yes	83.4	75.7,88.9	91.7	89.9,93.2	
Information provided about what to expect during labour and birth					0.58
Yes	91.3	85.0,95.2	93.3	91.6,94.7	
Information provided about what to support husband could provide					0.62
Yes	94.6	88.8,97.5	92.5	90.7,93.9	
Information about Community breast feeding resources					0.77
Yes	85.9	78.4, 91.1	83.3	80.8, 85.6	
Information about warning signs of complications					0.18
Yes	80.7	72.9, 86.7	86.5	84.3, 88.5	
Information about effects of medication on baby					0.35
Yes	91.5	85.2, 95.3	94.7	91.3, 95.9	
Information about SIDS*					0.000
Yes	80.3	72.5, 86.3	92.8	91.0, 94.2	
Information about use of car-seat by the baby					0.14
Yes	95.3	90.5, 97.7	94.0	92.4, 95.3	
Information about Postpartum depression					0.05
Yes	89.2	82.7, 93.5	94.0	92.4, 95.3	
Information about birth control after pregnancy					0.07
Yes	82.0	74.3, 87.7	88.8	86.7, 90.6	
Information about how to breastfeed the baby					0.03
Yes	95.4	89.8, 98.0	91.7	89.8, 93.3	
Information about formula feeding					0.001
Yes	92.7	86.8, 96.0	78.2	75.5, 80.7	

*Sudden Infant Death Syndrome.

**Table 3:** Comparison of pregnancy experiences between newcomer and Canadian-born women using weighted proportions

	**Newcomer (N = 140)**		**Canadian-born (N = 1137)**		**P-value**
	**Weighted mean**	**95% CI**	**Weighted mean**	**95% CI**	
**Perception of intrapartum care**					
Health care provider recommended C-section	16.8	11.3, 24.2	15.9	13.8, 18.3	0.58
Type of actual birth					0.02
Vaginal	63.9	55.2, 71.8	75.3	72.5, 77.8	
C-section	36.1	28.2, 44.8	24.7	22.2, 27.5	
Planned C-section					
Yes	47.3	33.0, 61.9	49.4	43.2, 55.6	0.86
Planned medical or non-medical reasons (for C-section)					0.03
Planned medical	*Over 80%		85.0	77.3, 90.4	
Non-medical	Suppressed		15.0		
**Exercise of choice**					
Was labour induced					0.1
Yes	50.3	40.1, 59.9	43.2	39.9, 46.5	
Use of forceps					0.03
Yes	13.1	7.5, 21.8	6.0	4.6, 7.9	
Use of stirups					0.01
Yes	70.1	58.8, 79.4	52.1	48.5, 55.7	
Episiotomy					0.35
Yes	19.5	12.8, 28.6	14.2	12.1, 16.6	
**Pain relief**					
Informed about labour progress					0.87
Yes	87.7	79.1, 93.1	89.0	86.7, 90.9	
**Post-natal care**					
Contacted by Public Health Nurse (PH visit)					0.31
Yes	*Over 97%	93.6, 99.1	99.1	98.4, 99.5	

Although not reported in the tables, family doctors and nurses were sources of information for both groups, but more so for Canadian-born women. Fifteen percent of Canadian-born women reported a family doctor as their source of information compared to 10% of newcomer women. Similarly, 20% of Canadian-born women reported a nurse as their source of information compared to 13% of newcomer women. Newcomer women were also less likely than Canadian-born to get information from friends and family (10% vs. 20%). They reported that books were a source of information more often than Canadian-born women (27% vs 17%).

On the whole there was no difference in reported levels of satisfaction between the two groups regarding types of information received, provider competency, provider concern for their privacy, provider respectfulness or their inclusion in decision-making (Table 4). The only difference in indicators of satisfaction was that fewer newcomers were “very satisfied” with health care received since birth compared to Canadian-born women (p = 0.03).

**Table 4:** Comparison of Satisfaction with Maternity Care between Newcomer and Canadian-born women using weighted proportions

	**Newcomer**		**Canadian-born**		**P-value**
	**n = 140**		**n = 1137**		
	**Weighted mean**	**95% CI**	**Weighted mean**	**95% CI**	
**Satisfaction with pregnancy care**					
Satisfied with information from providers					0.24
Very satisfied	57.8	49.1, 66.1	64.2	61.2, 67.1	
Somewhat satisfied	30.5	23.2, 39.0	27.7	25.0, 30.5	
*Indifferent or dissatisfied	11.7		8.1		
**Satisfied with provider competency**					0.52
Very satisfied	73.8	65.4, 80.7	77.7	75.1, 80.1	
Somewhat satisfied	21.6	15.3, 29.6	16.1	14.0, 18.5	
Indifferent or dissatisfied	4.6		6.1		
**Satisfied with provider concern for your privacy**					0.86
Very satisfied	74.8	66.5, 81.6	76.6	73.9, 79.1	
Somewhat satisfied	17.2	11.6, 24.7	15.8	13.7, 18.1	
Indifferent or dissatisfied	8.1		7.6		
**Satisfied with respect from provider**					0.79
Very satisfied	Over 97%**		79.2	76.6, 81.6	
Somewhat satisfied	Suppressed		15.4	13.4, 17.7	
Indifferent or dissatisfied	Suppressed		5.4		
Satisfied with say in decision-making					0.17
Very satisfied	67.1	58.4, 74.7	73.7	70.9, 76.3	
Somewhat satisfied	25.9	19.0, 34.2	20.6	18.2, 23.2	
Indifferent or dissatisfied	7.0		5.7		
**Overall satisfaction questions**					
**Satisfied with healthcare received since birth**					0.03
Very satisfied	56.1	47.4, 64.5	69.4	66.5, 72.2	
Somewhat satisfied	34.7	27.0, 43.4	21.2	18.8, 23.8	
Indifferent or dissatisfied	9.2		9.4		

*For those who were “indifferent” or “dissatisfied” the numbers were too small in either group to create meaningful CI’s and between group differences were only found for those “satisfied” or “somewhat satisfied”.

**Very satisfied and somewhat satisfied.

## Discussion

Access to and use of maternity services was compared between newcomer and Canadian-born women to evaluate potential barriers to system navigation and maternal satisfaction. Significant socio-demographic differences between newcomer and Canadian-born women were found, with newcomers more likely to be older, university educated, married, and residing in urban areas but with lower incomes. There were no differences between newcomer and Canadian-born women with respect to their ability to access maternity services, with all women reporting high levels of access. An area of concern is that newcomers were more likely to have assisted birth, either with forceps or by C-section and be placed in stirrups for birth. They were also less satisfied with the amount of information provided to them regarding a variety of important topics including physical and emotional changes that take place during pregnancy, Sudden Infant Death Syndrome, postpartum depression and infant feeding. They did, however, feel as informed as Canadian-born women regarding what to expect during birth and partner support, so it is a bit unclear what may underlie the differences since all these types of information are provided in the same pre-natal period.

Overall, our findings provide evidence that newcomer women to the Canadian Prairie Provinces were able to navigate the Canadian health care in a manner similar to Canadian–born women with respect to attaining prenatal care when they wanted it and attending an appropriate number of prenatal visits. This finding is inconsistent with those reported in a systematic review of 29 studies where it was concluded that migrant women were more likely to receive inadequate prenatal care [19]. However, most of the studies for this review were conducted in the US rather than Canada where healthcare is publicly funded. Thus, our finding is one that we did not find reported previously. It is worth mentioning that our research was limited by a small cross-sectional sample size so we cannot rule out a Type 1 errors based on the small amount of data, but on the whole we feel that our sample was robust in that between-groups differences emerged in expected areas. We explored over 40 variables, of which only 12 emerged as significant.

The finding that newcomer women were more likely to experience an assisted birth or deliver with their legs in stirrups is concerning. It is not clear if this finding is related to lack of communication between newcomer women and their providers or possibly an expectation based on their previous birth experiences. For example, lithotomy position with stirrups is a typical birth position in parts of the world and is considered part of routine care in many sub Saharan African countries including Zambia [20] and Ghana (personal communication (Florence Gans-Larty, Principal, Nursing & Midwifery Training School, Agogo, Ghana. 14th September 2012) so it is possible that some newcomers did not consider another position for birth. Very little recent existing evidence was found about the characteristics of women who were placed in stirrups to give birth. Only one study, using randomized controlled methodology (n = 108) explored the differences in perineal outcome comparing between use of stirrups and no stirrups [21]. In our study it was not clear whether newcomer women were advised about available choices. It is reasonable to assume that either newcomer women did not question the providers request to position themselves in the lithotomy position or if they did, they were not as assertive as their Canadian-born counterparts. It may also reflect the possibility that they did not have opportunities to obtain sufficient antenatal information regarding choices for position during birth.

Our findings that newcomer women have higher a C-section rate is consistent with existing evidence. For example, a systematic review and meta-analysis of 67 studies, showed significant differences in C-section rates between migrant and non migrant women in Western Industrialized countries with highest rates found in migrants from Sub Saharan Africa, Somalia and South Asia [22]. Low rates of C-section were found in newcomers from Eastern Europe and Vietnam. In another meta-analysis of national data from six countries, Somali newcomers (n = 10,431) were more likely to receive C-sections than country-born women. Using data from Medical Birth Registry of Norway data (n = 553,491), Vangen et.al showed that the frequency and indications for C-section were higher amongst immigrant groups compared ethnic Norwegians [23]. Little is however known why c-section rates are higher in newcomer groups. The authors of these studies hypothesized language/communication barriers, low social economic status, poor maternal health, GDM/high BMI and inadequate prenatal care as possible causes [22,23]. It is possible that newcomers are more reticent to challenge provider opinions or practices or do not have an opportunity to discuss options during antenatal visits. Further, if they have language challenges, they may not have the opportunity to make their preferences known whilst in labour. It has also been reported that women who have experienced female genital cutting may find providers more reluctant to manage a vaginal birth [24].

Newcomer women were less satisfied with information provided around infant feeding, including breastfeeding, and this could well contribute to fewer newcomers feeling “very satisfied” with their postnatal care. In many cultures family and close friends are important and trusted sources of perinatal information but they are less likely to be available to newcomer women. Further language differences between newcomers and their providers pose a barrier to accessing crucial information about pregnancy and navigating the healthcare system during pregnancy. It is also possible that what appears as lack of information maybe also be explained as a reluctance to accept practices that do not fit in with existing traditional and cultural beliefs during the perinatal period. For example, in a Canadian qualitative study, Grewal and colleagues [7] reported that the pervasiveness of perinatal traditional beliefs in Punjabi women remain relevant well after the time when they migrate to another country. In large parts of South Asia, postponing breast feeding for the first three days is a common practice due to a belief that colostrum is dangerous for the baby.

An important limitation of our analyses was the small sample size of newcomer women in the three Prairie Provinces on which we chose to focus. One consequence of the small sample size was an inability to use bootstrap analysis, which meant we could not take the design effect of the survey into account when calculating the estimators. This resulted in wider confidence intervals, making significant findings more difficult to interpret. It is also important to note that Canada is a huge and diverse country. Increases in migration to the Prairie Provinces is relatively recent so it cannot be inferred that migration patterns or findings would be similar in all regions of Canada.

## Conclusion

Canada is a country that has used immigration as a population expansion policy resulting in a highly multicultural society. The present research suggests that for the most part, maternity services to a diverse group of newcomer women as been managed. While there is room for improvement, this research suggests it might be prudent to continue with current policies. Areas that might benefit from a critical review are availability of choices in uptake of technologies such as C-sections or even simple things like birth position. It is critical health care providers ensure newcomers are provided information that they can understand.

While Canada has been reasonably successful in providing maternity services, the same cannot be said for socio-economic integration. Newcomer women were disproportionately more likely to have a university degree but a low income. This remains a challenge that needs to be addressed because while this inequity may be an important determinant of long term health and wellbeing, it can limit access to optimal health services. Further discussion on ways to address these disparities needs to be a part of a broader discussion that is beyond the scope of this paper.

## Consent

Statistics Canada, at the time of MES data collection, obtained informed consent from survey respondents that included a statement that the data they provide will be used for publications. We used the secondary MES dataset.

## Competing interests

The authors declare that they have no competing interests.

## Authors’ contributions

ZM was involved in the concept, design, statistical analysis and writing of the manuscript. BO was involved in the concept, design, statistical analysis and writing of the manuscript. BO is an original member of the working group of the Canadian Perinatal Surveillance Systems (CPSS) working on the Maternity Experiences Survey with the Public Health Agency of Canada and Statistics Canada. GH was involved with the concept and design of the study. All authors read and approved the final manuscript.

## Pre-publication history

The pre-publication history for this paper can be accessed here:

http://www.biomedcentral.com/1471-2393/14/4/prepub
